# Relationships between carotid artery intima-media thickness and echogenicity and body composition using a new magnetic resonance imaging voxel-based technique

**DOI:** 10.1371/journal.pone.0254732

**Published:** 2021-07-23

**Authors:** Lars Lind, Joel Kullberg, Håkan Ahlström, Robin Strand

**Affiliations:** 1 Department of Medical Sciences, Uppsala University, Uppsala, Sweden; 2 Division of Radiology, Department of Surgical Sciences, Uppsala University, Uppsala, Sweden; 3 Antaros Medical AB, BioVenture Hub, Mölndal, Sweden; 4 Department of Information Technology, Uppsala University, Uppsala, Sweden; Temple University School of Medicine, UNITED STATES

## Abstract

**Background:**

We evaluated how carotid artery intima-media thickness (IMT) and the echogenicity of the intima-media (IM-GSM), measured by ultrasound, were related to body composition, evaluated by both traditional imaging techniques, as well as with a new voxel-based “Imiomics” technique.

**Methods:**

In 321 subjects all aged 50 years in the POEM study, IMT and IM-GSM were measured together with a DXA scan for determination of fat and lean mass. Also a whole-body MRI scan was performed and the body volume was divided into >1 million voxels in a standardized fashion. IMT and IM-GSM were related to each of these voxels to create a 3D-view of how these measurements were related to size of each part of the body.

**Results:**

IM-GSM was inversely related to almost all traditional measurements of body composition, like fat and lean mass, liver fat, visceral and subcutaneous fat, but this was not seen for IMT. Using Imiomics, IMT was positively related to the intraabdominal fat volume, as well of the leg skeletal muscle in women. In males, IMT was mainly positively related to the leg skeletal muscle volume. IM-GSM was inversely related to the volume of the SAT in the upper part of the body, leg skeletal muscle, the liver and intraabdominal fat in both men and women.

**Conclusion:**

The voxel-based Imiomics technique provided a detailed view of how the echogenicity of the carotid artery wall was related to body composition, being inversely related to the volume of the major fat depots, as well as leg skeletal muscle.

## Introduction

Intima-media thickness (IMT) and the echogenicity of the intima-media (IM-GSM) are two arterial wall characteristics that usually are measured by ultrasound in the carotid artery. Both of these characteristics have been related to future cardiovascular events (IM-GSM mainly in an inverse fashion) [1,2].

Several previous studies have shown that a thick IMT is related to obesity [3–8], and especially abdominal obesity [9,10]. However, one study showed visceral adipose tissue (VAT) to be more closely related to IMT than the more commonly used waist circumference [11], while another study could not find that VAT was superior of BMI [4]. Yet another study indicated that the relationship between VAT and IMT might be sex-dependent [8].

There are only a few studies investigating the relationship between IM-GSM and obesity, but this measurement of echogenicity has been found to be inversely linked to BMI [12,13].

Thus, it is obvious that no consensus exists regarding the impact of ectopic fat distribution and IMT and that sex might be of major importance in this regard. We therefore undertook a sex-stratified investigation in a population-based sample of men and women all aged 50 years, the Prospective investigation of Obesity, Energy and Metabolism (POEM) study, in which standard body composition measurements were collected, as well as a whole-body dual-energy X-ray absorptiometry (DXA) scan and a whole-body magnetic resonance scan (MRI). Apart from quantification of VAT, abdominal subcutaneous adipose tissue (SAT), liver and pancreatic fat at MRI, we also applied a novel voxel-based technique, “Imiomics”, by which the body volume was divided into >1 million voxels in a standardized fashion [14]. Imiomics enables the analysis of relations between whole body MRI and non-imaging patient information at high spatial resolution (given by the image resolution), in a holistic manner. IMT and IM-GSM were related to each of the voxels to create a 3D-view of how these measurements were related to size of each part of the body.

The hypothesis tested was that the voxel-based analysis would provide more detailed information on how IMT and IM-GSM are related to body composition than traditional measurements.

## Materials and methods

### Sample

Data form the Prospective study on Obesity, Energy, and Metabolism (POEM) [15] was used in this analysis. Public population registers at the municipality of Uppsala, Sweden were used to recruit random men and women at the age of 50 from the general population. The invitation was done by mail and participants received their invitation one month after their 50th birthday. In total, 502 individuals took part in the study, which was performed between October 2010 and November 2016. The participation rate was 25.0% (502 of 2008). No exclusion criteria were used. The study was approved by the ethics committee at Uppsala University (No. 2009/057 and No. 2012/143), and the participants gave their written informed consent. Basic characteristics of the subjects are given in Table 1.

**Table 1:** Basic characteristics of the sample.

	Women		Men	
Variable	N	Mean (SD)	N	Mean (SD)
**Traditional risk factors**				
Systolic blood pressure (mmHg)	165	123.4 (17)	156	127.8 (15.9)
Diastolic blood pressure (mmHg)	165	74.2 (9.9)	156	79.1 (10.7)
Fasting glucose (mmol/l)	165	4.5 (.5)	156	4.6 (.8)
Serum cholesterol (mmol/l)	165	5.2 (.9)	156	5.4 (1.1)
LDL- cholesterol (mmol/l)	165	3.3 (.8)	156	3.5 (.9)
HDL- cholesterol (mmol/l)	165	1.5 (.3)	156	1.3 (.4)
Smoking (%)	165	4.4	156	14.6
**Vascular measurements**				
Intima-media thickness (IMT)(mm)	165	0.62 (.11)	156	.66 (.12)
Intima-media echogenicity (IM-GSM)	165	68.1 (15)	156	66.6 (15.3)
**Body composition measurements**				
Height (cm)	165	166.2 (6.6)	156	179.2 (6.3)
Weight (kg)	165	71.8 (12.6)	156	85.7 (11.9)
Waist circumference (cm)	165	89.6 (11.1)	156	94.5 (10)
Hip circumference (cm)	165	103.1 (8.4)	156	101.1 (6.3)
BMI (kg/m^2^)	165	26.0 (4.6)	156	26.7 (3.6)
Waist/hip-ratio	165	0.9 (.1)	156	0.9 (.1)
Total fat mass at DXA (kg)	154	26.6 (9.8)	148	21.8 (8.8)
Total lean mass (kg)	154	42.3 (4.7)	148	60.1 (5.9)
Fat mass at trunk (kg)	154	13.5 (5.5)	148	13.5 (5.7)
Fat mass at leg (kg)	154	9.5 (3.4)	148	5.7 (2.3)
Lean mass at leg (kg)	154	13.7 (1.8)	148	20.1 (2.3)
Fat mass at arm (kg)	154	2.8 (1.2)	148	1.9 (.9)
Lean mass at arm (kg)	154	4.6 (.7)	148	7.8 (1.1)
Liver fat (%)	138	3.4 (5.7)	121	5 (5.7)
Pancreas fat (%)	138	4.3 (3)	120	7.1 (6.4)
Visceral adipose tissue (L)	165	2.5 (1.5)	156	4.4 (2.2)
Subcutaneous adipose tissue (L)	165	7.6 (3.4)	156	5.7 (2.8)

The examination was performed after overnight fast. A calibrated mercury sphygmomanometer was used to measure blood pressure in the non-cannulated arm to nearest mmHg after at least 30 min of rest. The average of three recordings was used. Standard laboratory techniques were used to measure lipid variables and fasting blood glucose. From these risk factors and information on medications, smoking and prevalent diabetes, the Framingham score was calculated [16]. Waist and hip circumference were recorded at the umbilical and trochanter levels, respectively, and WHR was calculated. Fat and lean mass was established using DXA. Whole-body MRI and dedicated imaging of liver and pancreas were performed on those who volunteered for this part of the study. MRI was performed on a separate day, within one month from the main study visit. This study includes only the 326 subjects with a technically appropriate MRI registration.

### Carotid artery ultrasound

The carotid artery was assessed by external B-mode ultrasound imaging (Acuson XP128 with a 10 MHz linear transducer, Acuson Mountain View, California, USA). The common carotid artery (CCA), the bulb and the internal carotid artery (ICA) were visualized (12).

The images were digitized and imported into the AMS (Artery Measurement Software) automated software for dedicated analysis of intima-media thickness (IMT) and the grey scale median of the intima-media complex. The IMT was evaluated in the far wall in the CCA 1–2 cm proximal to the bulb. A maximal 10 mm segment with good image quality was chosen for IMT-analysis. The program automatically identifies the borders of the IMT of the far wall and the inner diameter of the vessel and calculates IMT from around 100 discrete measurements through the 10 mm long segment. This automated analysis could be manually corrected if not found appropriate at visual inspection. The given value for carotid artery IMT is the mean value from both sides.

A region of interest was placed manually around the intima-media segment that was evaluated for intima-media thickness and the program calculates the echogenicity in the intima-media complex from analysis of the individual pixels within the region of interest on a scale from 0 (black) to 256 (white). The blood was used as the reference for black and the adventitia was the reference for white. It is assumed that a low value indicates lipid infiltration. The grey-scale median (GSM)-value given is the mean value from both sides (IM-GSM).

The measurements were repeated in 30 random subjects giving a coefficient of variation of carotid artery IMT of 7.2% and 7.5% for IM-GSM.

### DXA

Total and regional body fat and lean mass were estimated using the same Dual-energy X-ray absorptiometry scanner (DXA; Lunar Prodigy, GE Healthcare). To minimize potential operator bias, one experienced nurse performed all scans in the same room. The precision error of the DXA measurements in our laboratory was calculated using triple measurements in 15 subjects with repositioning according to recommendations from the International Society for Clinical Densitometry. Total fat and lean mass evinced a precision error of 1.5% and 1.0%, respectively. In the analysis, automatic edge detection was consistently employed; nevertheless, all scans were carefully checked for errors and manually corrected if necessary.

### MRI

A continuously moving bed setup was used to image subjects in supine position with the integrated body coil using a 1.5T clinical MR system (Philips Achieva, Philips Healthcare, Best, Netherlands). The whole-body water-fat image data was acquired using a spoiled 3D multi gradient echo sequence. The following scan parameters were used: TR/TE1/ΔTE = 5.9/1.36/1.87 ms, flip angle three degrees, three unipolar echoes. The field of view (FOV) was 530×377×2000 mm^3^, and the reconstructed voxel size was 2.07×2.07×8.0 mm^3^ in left-right×anterior-posterior×foot-head directions. In addition to the whole-body scan, a separate scan of the liver, also including the pancreas, was undertaken. This dedicated scan was used for detailed analysis of liver and pancreas fat content. The first 94 subjects in the POEM study did not undergo the liver scan. The liver scan parameters were: TR/TE1/ΔTE = 8.66/0.92/1.32, flip angle five degrees, six unipolar echoes. The imaged FOV was 384×288×150 mm^3^, and the reconstructed voxel size was 3.0×3.0×10.0 mm^3^. An inhouse developed algorithm was used for the water–fat image reconstruction. Both the imaging protocol and the reconstruction method have previously been described in more detail [17]. Quantification of liver and pancreas fat was performed by manual volume of interest delineation in the software ImageJ (version 1.45s). As much as possible of the volume of interest was delineated by a trained operator. Tissue borders were avoided in order to limit partial volume effects. To get the measurements of tissue fat content, the median fat content was computed in the volumes of interest.

The VAT and SAT depots were quantified by an atlas-based segmentation approach; manually defined VAT and SAT depots in a male and female reference subject were deformed to all other subjects by utilizing the same image-registration method that was used for the Imiomics analysis, see the Section *Imiomics*. Further processing of the deformed regions included thresholding operations and excluding voxels with fat content <50%.

### Imiomics

The Imiomics technique is based on image registration, in which a deformation field is computed and applied on a target image to match a (fixed) reference image. The deformation field matches each point in the reference image with the corresponding point in the target image. The point-to-point correspondences are obtained in the Imiomics analyses by deforming whole-body MRI images to a reference whole-body MRI volume. In this way, the reference whole-body MRI volume acts as a reference coordinate system—each point in the reference volume has a corresponding point in all volumes in the cohort. By utilizing the point-to-point correspondences, a voxel-wise statistical analysis procedure, in which associations between MRI fat content and tissue volume can be related to study non-imaging data, is enabled [18].

It is crucial for the Imiomics analyses that the registration method is robust. This has been achieved by employing a tissue-specific handling of bone, lean tissue and adipose tissue. The degree of elasticity (the ability to stretch) of deformations required to align images tends to differ across these different types of tissues. By performing the image registration of the different tissues sequentially and with different registration parameters, this prior information can be utilized. The following steps are followed by the Imiomics image registration method: 1) Bone sections registration: Articulated, piece-wise affine, registration; 2) Water image registration: Semi-elastic registration with constraints on bone; 3) Fat image registration: Elastic registration with constraints on bone and water. The image registration method has previously been shown to produce image registration results appropriate for Imiomics analyses. Ekström et al. presented and evaluated [19] the registration method on MRI water–fat image data.

### Statistics

Linear regression analysis (OLS) was used to evaluate the relationships between traditional measurements of anthropology and fat distribution and IMT and IM-GSM. The relationships are presented as regression coefficients and were stratified by sex. Variables that were skewed to the right were ln-transformed to achieve a normal distribution (liver fat, pancreatic fat, VAT, SAT). Measurements of anthropology and fat distribution were transformed to a SD-scale in order to improve the comparison between these indices. The analyses were performed unadjusted, as well as adjusted for systolic blood pressure, LDL- and HDL-cholesterol, diabetes and smoking (included in the Framingham score).

STATA 16 was employed for these computations. (Stata Corp, College Station, TX).

For the Imiomics analyses, Spearman rank coefficient correlations and partial correlations were computed to generate the voxel-wise ρ- and r-values, respectively, for all parameters. For significance level, p-values were also computed. MATLAB (version 2016b, The MathWorks Inc., Natick, MA) was employed for these calculations. The visualizations show so called correlation-maps where each voxel’s correlation value and p-value are visualized in the following way: Voxels with p-value > = 0.05 are transparent and the water content is shown. Voxels with p-value <0.05 (“significant”, uncorrected) show the corresponding correlation coefficient in the colormap *jet* inverted, where red represents strong positive correlation and blue represents strong negative correlation.

The Imiomics results were analyzed visually. To minimize the number of false positive findings from the large number of tests performed, we report only associations found in many image elements within a certain anatomical structure, corresponding to p-values far below 0.05.

## Results

### IMT vs traditional measurements

Of all evaluated traditional measurements of body composition, IMT was only related to lean mass at borderline significance (Table 2).

**Table 2:** Relationships between intima-media thickness (IMT) and traditional measurements of body composition.

	Male								Female							
	Unadjusted				Adjusted				Unadjusted				Adjusted			
	Beta	95%CI lower	95%CI higher	p-value	Beta	95%CI lower	95%CI higher	p-value	Beta	95%CI lower	95%CI higher	p-value	Beta	95%CI lower	95%CI higher	p-value
Height	.2	-.03	.43	.08	.17	-.06	.4	.13	.18	-.02	.38	.08	.17	-.03	.37	.10
Weight	.16	-.03	.35	.08	.1	-.12	.32	.39	.15	-.01	.32	.06	.05	-.13	.23	.56
Waist circumference	.08	-.1	.25	.37	-.01	-.2	.19	.92	.12	-.02	.27	.08	.03	-.12	.19	.67
Hip circumference	.1	-.09	.29	.31	.04	-.17	.25	.71	.05	-.08	.19	.42	-.02	-.16	.11	.73
BMI	.07	-.11	.26	.43	-.01	-.21	.2	.95	.07	-.06	.21	.28	-.01	-.16	.13	.85
Waist/hip-ratio	.06	-.14	.25	.57	-.05	-.26	.16	.64	.15	-.01	.3	.06	.08	-.09	.24	.34
Total fat mass at DXA	.03	-.15	.21	.75	-.05	-.25	.15	.62	.09	-.06	.24	.24	0	-.16	.16	.97
Total lean mass	.32	.04	.6	.02	.3	0	.6	.047	.36	.02	.7	.03	.28	-.07	.64	.11
Fat mass at trunk	.03	-.13	.19	.72	-.05	-.23	.13	.58	.1	-.05	.26	.20	.01	-.16	.18	.90
Fat mass at leg	-.02	-.27	.22	.85	-.09	-.35	.16	.48	.06	-.1	.22	.45	-.01	-.17	.15	.88
Lean mass at leg	.27	0	.53	.047	.24	-.04	.52	.09	.27	-.05	.6	.09	.17	-.16	.51	.30
Fat mass at arm	.12	-.09	.32	.26	.07	-.16	.29	.55	.1	-.05	.25	.17	.01	-.15	.17	.91
Lean mass at arm	.3	.03	.58	.03	.28	0	.55	.046	.42	0	.85	.051	.33	-.12	.77	.14
Liver fat	.14	-.04	.32	.12	.11	-.09	.31	.27	.16	-.01	.33	.07	.11	-.09	.31	.29
Pancreas fat	.02	-.15	.19	.80	.01	-.17	.19	.90	.13	-.06	.32	.17	.1	-.1	.3	.31
Visceral adipose tissue	.1	-.08	.29	.26	.02	-.18	.23	.82	.14	-.01	.3	.07	.04	-.14	.22	.64
Subcutaneous adipose tissue	.07	-.08	.23	.35	-.01	-.18	.17	.94	.1	-.07	.27	.23	-.03	-.21	.15	.74

The analyses were stratified by sex and performed unadjusted (age 50 in all subjects), as well as adjusted for systolic blood pressure, LDL- and HDL-cholesterol, diabetes and smoking.

### IM-GSM vs traditional measurements

**Fig 1:**
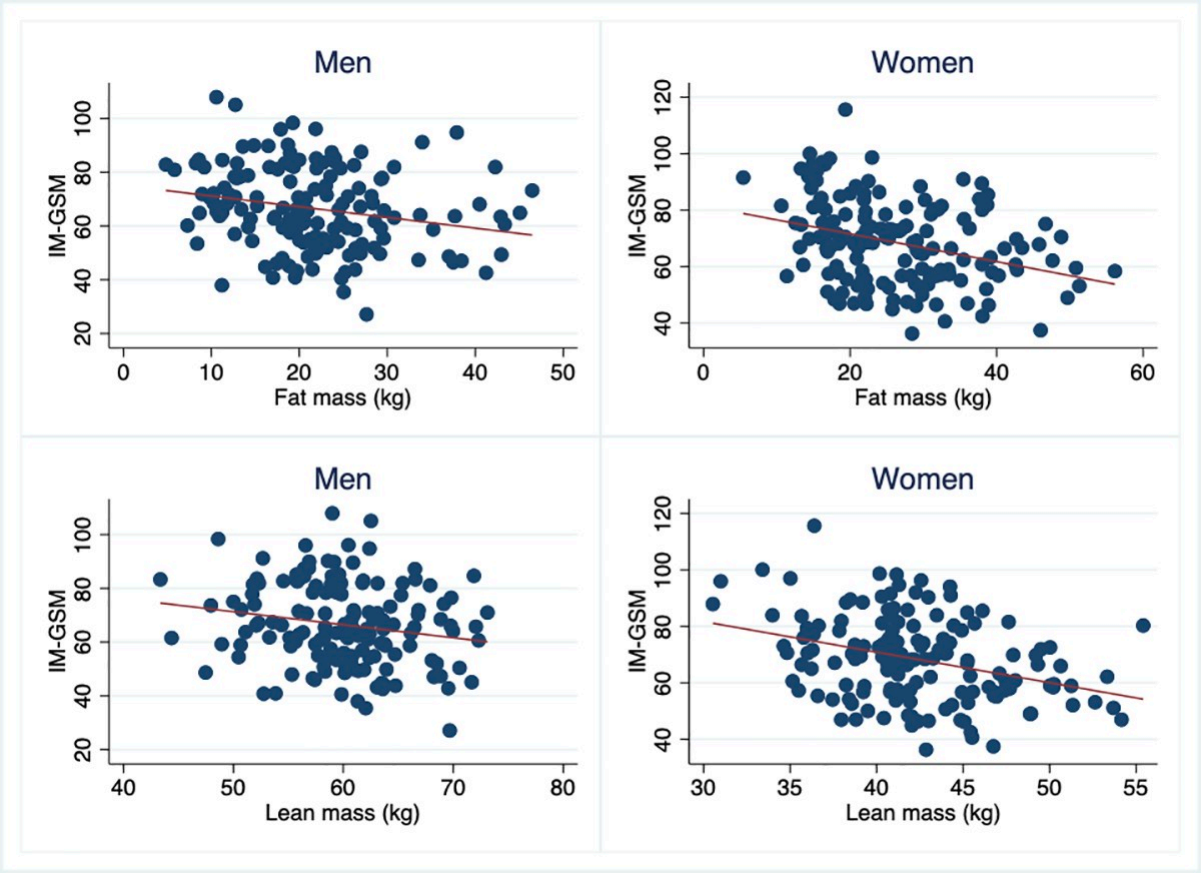
Relationships between the echogenicity of the intima-media (IM-GSM) and fat mass and lean mass in men and women.

IM-GSM was inversely related to almost all traditional measurements of body composition, except height. These relationships were generally stronger in women compared to men, and most were still significant, although slightly attenuated, following adjustment for traditional CV risk factors (Fig 1, Table 3).

**Table 3:** Relationships between echogenicity of the intima-media complex (IM-GSM) and traditional measurements of body composition.

	Male								Female							
	Unadjusted				Adjusted				Unadjusted				Adjusted			
	Beta	95%CI lower	95%CI higher	-p-value	Beta	95%CI lower	95%CI higher	-p-value	Beta	95%CI lower	95%CI higher	-p-value	Beta	95%CI lower	95%CI higher	-p-value
Height	-.04	-.27	.19	0.73	-.01	-.24	.23	0.95	-.1	-.31	.11	0.35	-.13	-.34	.08	0.21
Weight	-.32	-.51	-.14	.0005	-.36	-.57	-.14	.001	-.38	-.54	-.21	4.5e-06	-.32	-.5	-.15	.0003
Waist circumference	-.26	-.43	-.09	.002	-.26	-.45	-.07	.008	-.29	-.43	-.15	.00005	-.23	-.39	-.07	.004
Hip circumference	-.28	-.47	-.09	.003	-.29	-.5	-.09	.005	-.23	-.36	-.1	.0005	-.19	-.33	-.05	.006
BMI	-.31	-.49	-.14	.0005	-.33	-.53	-.13	.001	-.28	-.41	-.15	.00002	-.23	-.37	-.08	.002
Waist/hip-ratio	-.21	-.41	-.02	.03	-.17	-.38	.04	.10	-.23	-.39	-.07	.004	-.14	-.31	.03	.11
Total fat mass at DXA	-.25	-.43	-.08	.005	-.24	-.44	-.04	.01	-.31	-.46	-.17	.00002	-.24	-.4	-.09	.002
Total lean mass	-.33	-.62	-.05	.02	-.33	-.63	-.02	.03	-.75	-1.07	-.42	9.2e-06	-.67	-1.01	-.32	.0001
Fat mass at trunk	-.24	-.4	-.08	.003	-.24	-.42	-.05	.01	-.37	-.52	-.22	1.0e-06	-.3	-.47	-.14	.0003
Fat mass at leg	-.22	-.47	.03	.07	-.18	-.44	.08	.17	-.21	-.36	-.05	.009	-.14	-.3	.01	.07
Lean mass at leg	-.25	-.52	.02	.06	-.24	-.52	.05	.10	-.62	-.94	-.3	.0001	-.51	-.84	-.18	.002
Fat mass at arm	-.35	-.55	-.15	.0006	-.35	-.57	-.13	.001	-.33	-.47	-.19	5.4e-06	-.26	-.41	-.1	.001
Lean mass at arm	-.4	-.67	-.12	.005	-.38	-.66	-.11	.006	-.74	-1.17	-.32	.0006	-.58	-1.02	-.14	.009
Liver fat	-.13	-.32	.06	.17	-.09	-.3	.12	.39	-.24	-.42	-.07	.005	-.2	-.4	.01	.05
Pancreas fat	-.13	-.31	.04	.14	-.1	-.28	.08	.27	-.29	-.48	-.11	.002	-.24	-.43	-.04	.01
Visceral adipose tissue	-.31	-.49	-.14	.0005	-.32	-.52	-.12	.001	-.41	-.56	-.26	9.6e-08	-.36	-.53	-.18	.00006
Subcutaneous adipose tissue	-.23	-.39	-.08	.002	-.23	-.41	-.06	.009	-.36	-.52	-.2	.0001	-.28	-.47	-.1	.002

The analyses were stratified by sex and performed unadjusted, as well as adjusted for systolic blood pressure, LDL- and HDL-cholesterol, diabetes and smoking.

### Imiomics for IMT

**Fig 2:**
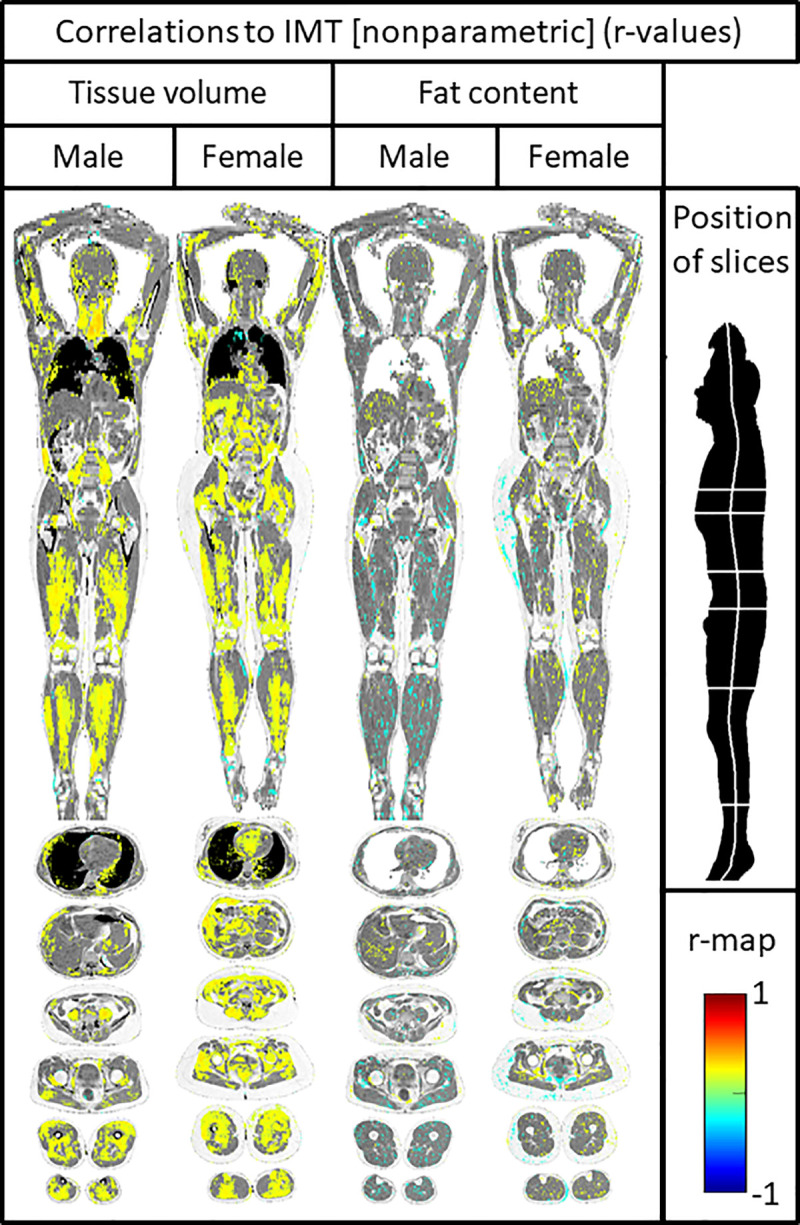
Imiomics correlation maps showing relations between tissue volume/fat content and carotid artery intima-media thickness (IMT). Significant (P<0.05) voxelwise non-parametric Spearman rank coefficient correlation values are shown in the colorscale in the lower right. Pixels with non-significant correlations show the underlying water signal values. One Coronal slice and six axial slices are shown, as given by the illustration in the upper right part of the figure.

In women, IMT was positively related to intraabdominal fat volume, as well of the leg skeletal muscle. Also, positive correlations with the blood volume in the heart was noted, indicating myocardial dilatation. In males, IMT was mainly positively related to the leg skeletal muscle volume (Fig 2).

Regarding the tissue lipid content, no strong relationships vs IMT could be seen neither in women, nor in men (Fig 2).

### Imiomics for IM-GSM

**Fig 3:**
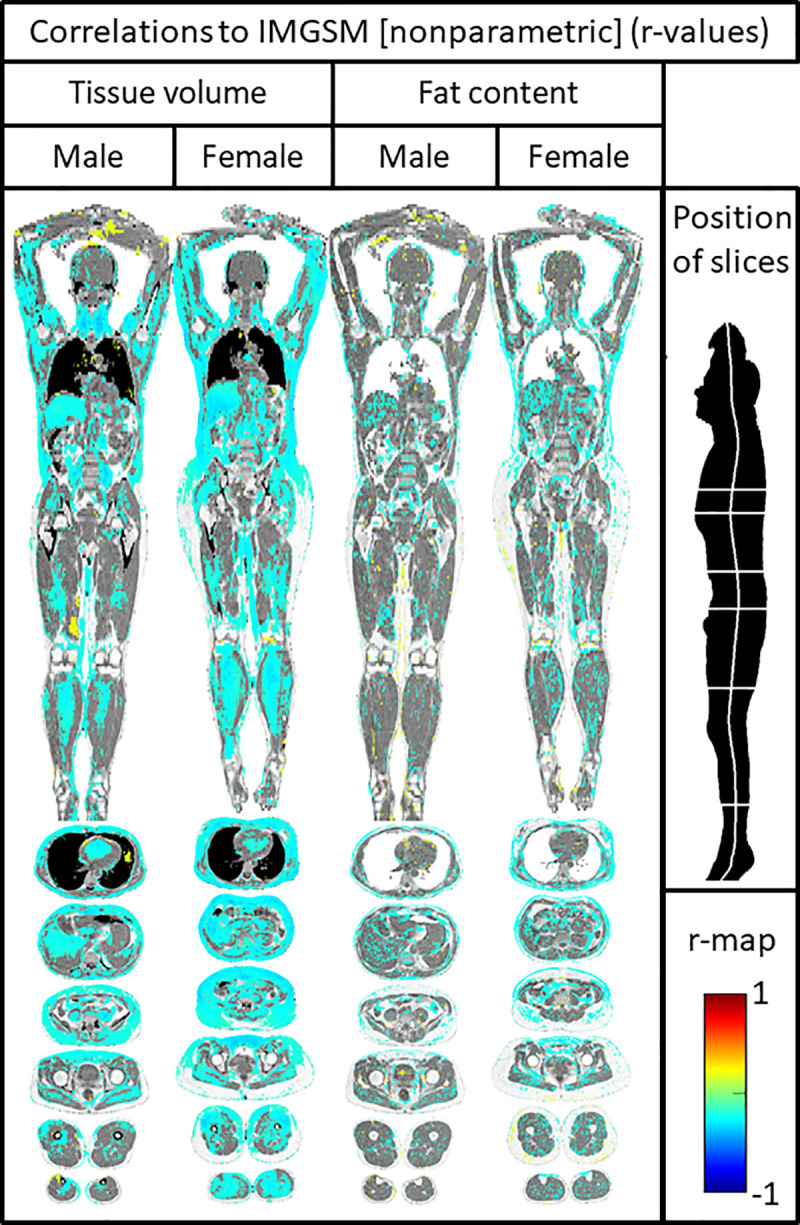
Imiomics correlation maps showing relations between tissue volume/fat content and the echogenicity of the intima-media (IM-GSM). Significant (P<0.05) voxelwise non-parametric Spearman rank coefficient correlation values are shown in the colorscale in the lower right. Pixels with non-significant correlations show the underlying water signal values. One Coronal slice and six axial slices are shown, as given by the illustration in the upper right part of the figure.

In women, IM-GSM was inversely related to the SAT volume in the upper part of the body, as well as inversely related to the volume of the lever and intraabdominal fat. IM-GSM was furthermore inversely related to the leg skeletal muscle volume and the blood volume in the heart. A similar picture was seen in males (Fig 3).

Regarding lipid content, in women, inverse relationships were seen vs SAT in the upper part of the body, liver and intraabdominal fat, as well as vs leg skeletal muscle. In men, inverse relationships were seen mainly vs liver fat (Fig 3).

**Fig 4:**
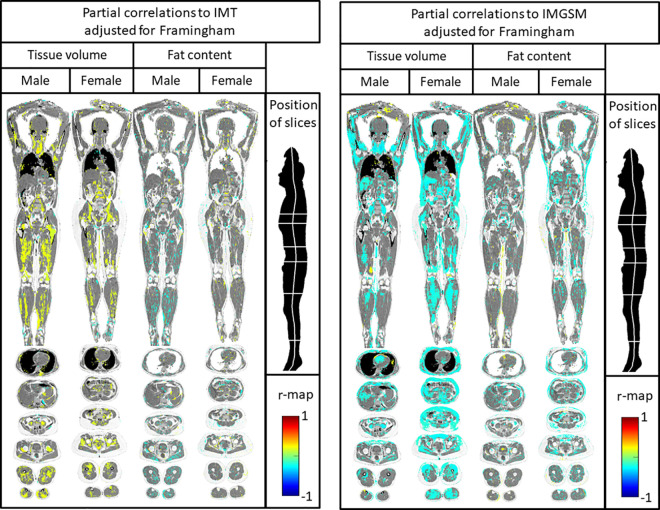
Imiomics correlation maps showing relations between tissue volume/fat content and carotid artery intima-media thickness (IMT) (left panel) and the echogenicity of the intima-media (IM-GSM) (right panel), when corrected for Framingham risk score. Significant (P<0.05) voxelwise partial parametric correlation values, with Framingham risk score as the confounding variable, are shown in the colorscale in the lower right. Pixels with non-significant correlations show the underlying water signal values. One Coronal slice and six axial slices are shown, as given by the illustration in the upper right part of the figure.

The above mentioned relationships for Imiomics were still significant, although slightly attenuated, when adjusted for the Framingham score (Fig 4).

## Discussion

The present study showed generally stronger relationships between IM-GSM and different body composition measurements than seen for IMT. These relationships were most often slightly stronger in women compared to men. The novel voxel-based technique provided a more detailed information than standard imaging methods.

### IMT and body composition

Much to our surprise, IMT was not significant related to any of the traditional obesity measurements, like BMI or waist circumference, in any of the sexes. IMT was however significantly related to blood pressure and LDL-cholesterol in the expected manner (p<0.001 and p = 0.008, respectively).

IMT was only related to lean mass at DXA of the traditional body composition variables. This finding is supported by a relationship vs expanded leg skeletal muscle by the voxel-based Imiomics technique. This was seen in both men and women. Similar findings have previously been reported using DXA [20,21]. The reason behind this finding is not clear. A large skeletal muscle mass is linked to a large cardiac output with an enlarged heart, dilated arteries and changed blood flow conditions in arteries, such as the carotid artery. If this could induce thickening of the intima-media complex is however not known, but the idea that hemodynamic factors could of importance is supported by the finding of a relationship between IMT and blood volume in the heart when using the voxel-based method.

The voxel-based method also disclosed a relationship between intra-abdominal fat and IMT in women, something that was not evident when quantifying VAT. Thus, the Imiomics technique is more sensitive to disclose relationships, as we previously have been demonstrating for other characteristics, like CV risk factors and endothelial function [22,23].

### IM-GSM and body composition

In sharp contrast to IMT, IM-GSM was inversely related to almost all traditional measurements of body composition in a powerful fashion. These relationships were generally stronger in women compared to men, and most were still significant following adjustment for traditional CV risk factors. A similar picture emerged when the voxel-based method was used, but using this new technique it could be seen that IM-GSM was only related to SAT in the upper part of the body. The relationship is not seen distal of the trochanter region. A strong relationship vs volume and lipid content in the ventral part of the lower abdomen was seen, as previously noted also for the relationship vs the metabolic syndrome [22].

Some of those findings were supported by DXA. Using DXA, arm fat mass was more closely related to IM-GSM than leg fat mass. However, the voxel-based method provides more detailed information.

Using the absolute fat content intensity values generated by the water-fat MRI protocol, it was also found that IM-GSM was inversely related to the lipid content in the leg skeletal muscle, not only the size. Increased intra-muscular fat content is a hallmark of peripheral insulin resistance [24], and we have previously shown IM-GSM to be related to insulin resistance measured by the gold standard hyperinsulinemic euglycemic clamp method [13].

Since IM-GSM previously have been found to inversely to CV mortality independently of IMT (1), it is of clinical value to evaluate if body composition is related to IM-GSM. Interesting relationships were found already with the traditional techniques, but the voxel-based methods provided further information on these relationships, such as the distribution of SAT and the intra-muscular fat content. The clinical usefulness of this new technique could however only be firmly evaluated in terms of CV disease when we perform a longitudinal study evaluating the findings obtain by imiomics in relation to future incident CV events.

### Strength and limitations

The major strength of the present study is that we have evaluated body composition with a couple of traditional imaging techniques, as well as the newly developed and validated voxel-based Imiomics technique [14,18]. This was performed in a fairly large sample which allowed for the important sex-stratification.

To cut down on the computer-intensive calculations for Imiomics, we adjusted for Framingham risk score rather than for the different risk factors included in the score.

Like with all other homogenous epidemiological samples, the findings have to be reproduced in other geographical and ethnical groups.

### Conclusion

IMT was not strongly related to different fat depots, but mainly to skeletal muscle mass. The voxel-based Imiomics technique provided a detailed view of how the echogenicity of the carotid artery wall was related to body composition, being associated with a reduction of the major fat depots, as well as leg to skeletal muscle lipid content and mass.
